# Atypical exophytic liver mass: Giant pedunculated hepatic haemangioma masquerading as a gastrointestinal stromal tumour of the gastric wall

**DOI:** 10.4102/sajr.v23i1.1697

**Published:** 2019-03-18

**Authors:** Venkatram Krishnan, Sunil K. Bajaj, Abhilash Sethy, Neetika Gupta

**Affiliations:** 1 Department of Radiodiagnosis, Safdarjung Hospital, Delhi, India

## Abstract

Haemangioma is the most common benign tumour of the liver. However, an exophytic hepatic haemangioma, especially the pedunculated form, is very rare. Giant pedunculated haemangiomas are prone to complications because of the narrow size of the pedicle. A large number of lesions can potentially present as exophytic liver masses, and accurate diagnosis can sometimes be challenging. We report a case of an incidentally discovered asymptomatic giant pedunculated liver haemangioma in the region of the lesser sac in a prospective renal donor, which was initially suspected to be a gastrointestinal stromal tumour of the stomach wall. Multiphasic computed tomography and confirmatory magnetic resonance imaging scans ultimately revealed the true nature of the lesion, which turned out to be an exophytic pedunculated hepatic haemangioma from the left lobe of the liver. The lesion was then surgically resected and histopathologically confirmed to be a haemangioma. The patient subsequently underwent successful renal donation as planned. Being a benign lesion with characteristic imaging features, accurate radiological diagnosis is absolutely essential for the appropriate management of such atypical haemangiomas.

## Introduction

Haemangioma is the most common benign tumour of the liver. The prevalence is about 0.4% – 20% in necropsies. Haemangiomas are usually solitary, occur more commonly in women and are usually found in the posterior segments of the right lobe of the liver. In most cases, they are small and asymptomatic and found incidentally on imaging studies.^1^ Some disagreement exists on the size criteria, but generally haemangiomas >10 cm are considered giant haemangiomas.^2,3^ Giant haemangiomas can be symptomatic secondary to mass effect on the abdominal viscera and surrounding structures.

Exophytic hepatic haemangiomas, especially the pedunculated forms, are very rare; only a few cases of these have been reported in literature.^2,3^ In almost half of the reported cases, pedunculated haemangiomas were found to be symptomatic at diagnosis.^1^ Giant pedunculated haemangiomas are even more likely to be symptomatic and are usually diagnosed because of complications secondary to the size of the lesion and its narrow pedicle. Asymptomatic cases frequently put forth a challenge in the radiological diagnosis because of difficulty in defining the origin of the mass, as the thin pedicle may sometimes be radiologically undetectable.^1^

Here we present a case of a middle-aged female patient who was being evaluated with multidetector computed tomography (MDCT) renal angiography as a prospective renal donor. A giant pedunculated haemangioma from the left liver lobe was incidentally discovered and thought to be a gastrointestinal stromal tumour (GIST) from the gastric wall. This lesion presented a diagnostic dilemma.

## Case report

A 51-year-old healthy asymptomatic female underwent MDCT renal angiography as a prospective renal donor. A large, well-defined, heterogeneously hypoattenuating mass lesion, 11.20 cm in the largest dimension, was incidentally discovered in the region of the lesser sac on the non-contrast computed tomography scan (NCCT). The origin of the lesion could not be delineated on a non-contrast scan, as it did not show a definite claw sign with any of the intra-abdominal organs. It was seen to abut and scallop the left lobe of the liver medially and abut the lesser curvature of the stomach posterolaterally; however, there was no obvious infiltration of the lesion into any of the adjacent organs. No significant regional lymphadenopathy or ascites could be identified. With an initial suspicion of a GIST arising from the stomach wall, MDCT renal angiography was performed in the arterial, portovenous and delayed (5 min) phases, with inclusion of the entire lesion in the scan plane.

**FIGURE 1:**
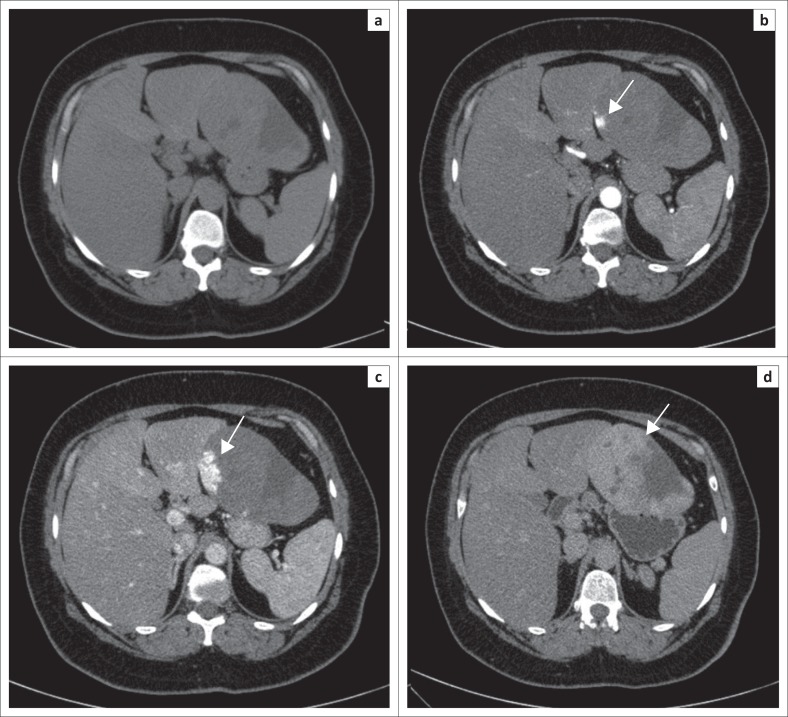
Non-contrast computed tomography scan (a) showing a mass lesion which appears separated from the liver and is causing scalloping of the left lobe, closely abutting the lesser curvature of the stomach and appearing inseparable from it. Post-contrast arterial phase (b) shows patchy peripheral enhancement adjacent to the left lobe of the liver (arrow). Portovenous (c) and delayed (d) phases show progressive filling in of the lesion with contrast (arrows). The pattern of enhancement is suggestive of a haemangioma.

**FIGURE 2:**
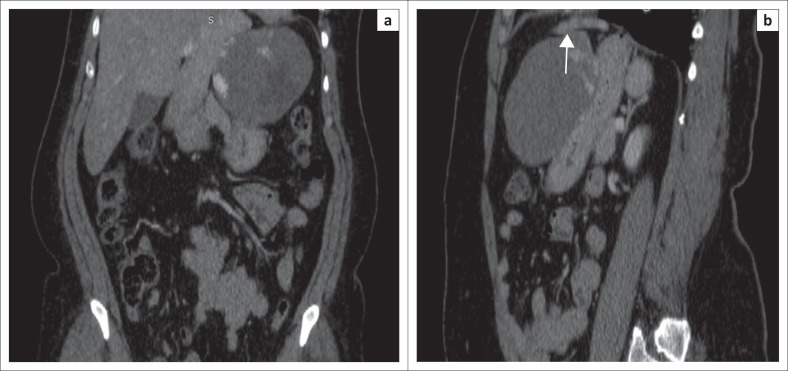
(a) Coronal reformat image shows the mass scalloping of the left lobe of the liver. (b) Sagittal reformat image shows the mass abutting a wide region of the lesser curvature of the stomach and appearing inseparable from it. However, close inspection shows a sliver of tissue (arrow) extending between the left lobe of the liver and the mass.

**FIGURE 3:**
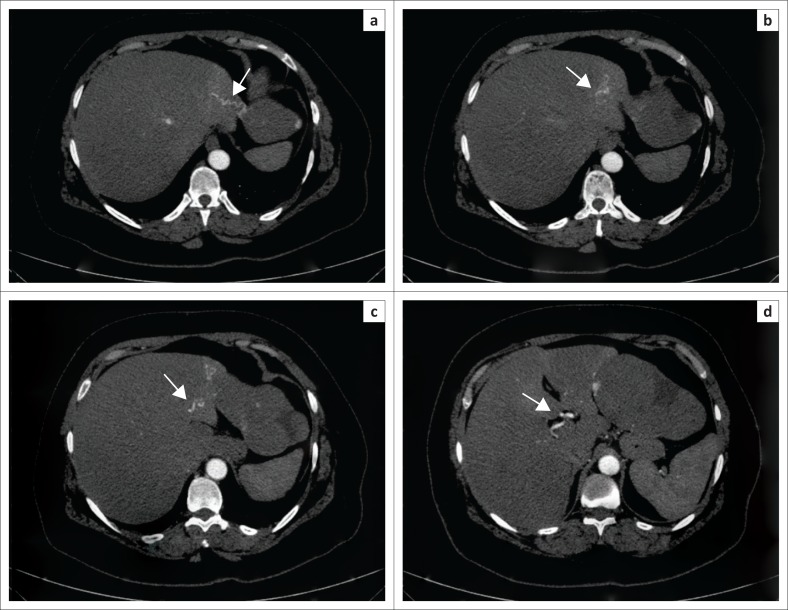
Arterial phase thin section reconstructions of the upper part of the mass (a) and (b) show a tortuous vessel with arterial flow within (arrow) passing from the left lobe of the liver into the mass through a thin sliver of tissue (pedicle). The adjoining part of the liver parenchyma also shows abnormal enhancement in the arterial phase. The vessel can be traced back to the left branch of the hepatic artery in successive images (c) and (d) (arrows).

The lesion in question showed patchy peripheral post-contrast enhancement in the arterial phase matching the aortic attenuation, predominantly in the superomedial juxtahepatic region of the mass. Progressive filling in of the mass with contrast was noted in the venous and delayed phases with a few non-enhancing areas within it (Figure 1). On multiplanar reconstructions, the relationship of the mass to the adjacent organs was assessed and no definite claw sign was observed with any of them (Figure 2). On close inspection, a few definite arterial vascular channels were identified from the left lobe of the liver passing into the mass through a sliver of tissue (pedicle) and supplying the mass in its uppermost part just subjacent to the diaphragm (Figure 3).

**FIGURE 4:**
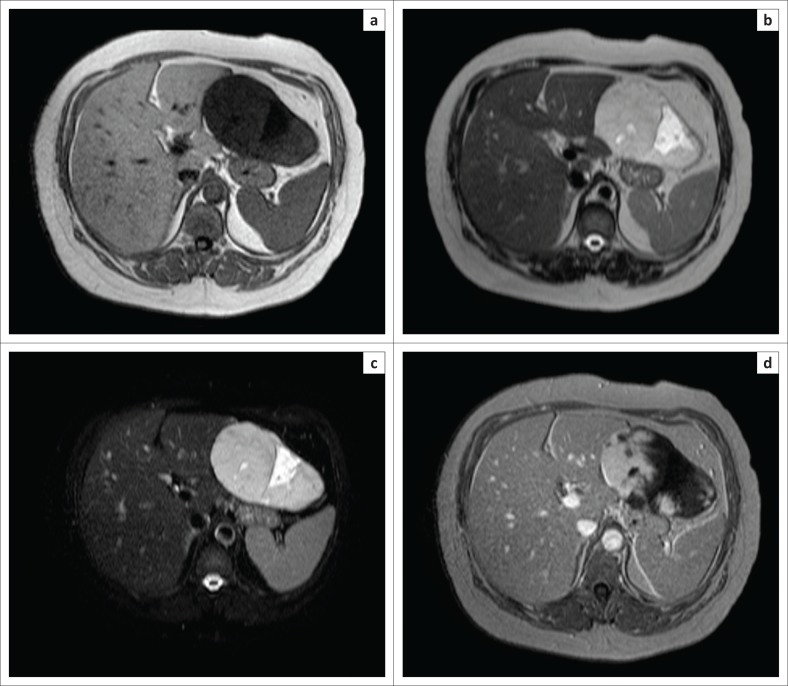
T1W image (a) reveals the mass to be hypointense with a cleft-like area of lower signal intensity within. The mass is brightly hyperintense on T2W (b) and SPAIR (c) suggestive of a light bulb sign with a cleft-like area of marked high signal intensity within signifying a hepatic haemangioma. Post-contrast scan (d) shows patchy peripheral enhancement. Dynamic contrast-enhanced scan (not shown) revealed centripetal enhancement with contrast from the arterial through the delayed phases.

On account of the importance of an accurate diagnosis in this patient in view of clearance for a renal transplant, magnetic resonance imaging (MRI) was performed in order to confirm the diagnosis. The mass appeared hypointense on T1 with a cleft-like area of lower signal intensity within and diffusely hyperintense on T2/T2 SPAIR (Spectral Presaturation Attenuated Inversion Recovery sequence: fat saturation sequence) (light bulb sign) with a cleft-like area of markedly higher signal intensity within. Further, dynamic contrast-enhanced MRI was performed which showed a similar pattern of enhancement in the arterial, portovenous and delayed phases as seen on MDCT angiography (Figure 4). The central cleft-like region did not show any enhancement.

**FIGURE 5:**
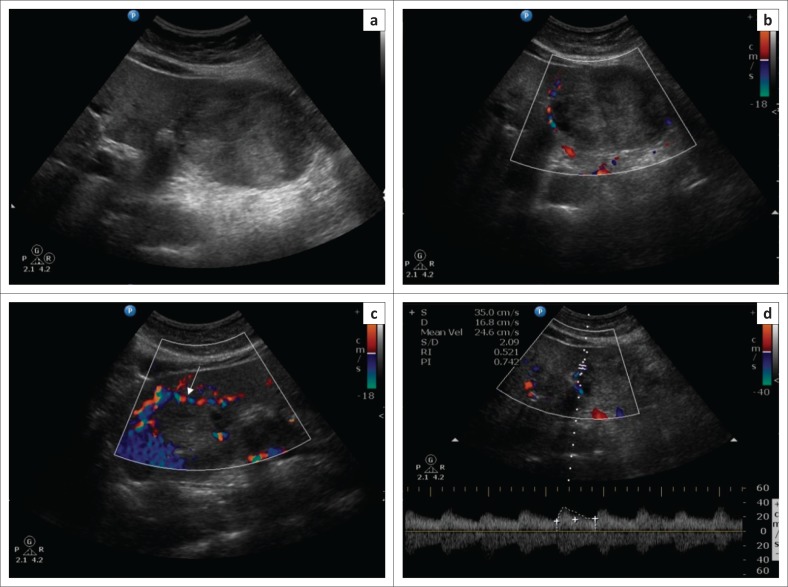
Grayscale ultrasound (a) shows the mass lesion scalloping the left lobe of the liver. Doppler examination (b) shows increased vascularity in the left lobe of the liver adjacent to the lesion with a tortuous bridging vessel (c) passing through a sliver of tissue from the left lobe to the mass (arrow). Doppler waveform (d) from the bridging vessel shows arterial flow.

Ultrasound Doppler scan was performed in order to further correlate the findings and confirm the flow pattern within the vessels. The bridging vessels between the liver and the mass were identified, with detectable arterial flow within them (Figure 5). A final diagnosis of an incidental asymptomatic giant pedunculated haemangioma of the left lobe of the liver with a thin vascular pedicle was made based on the imaging findings.

**FIGURE 6:**
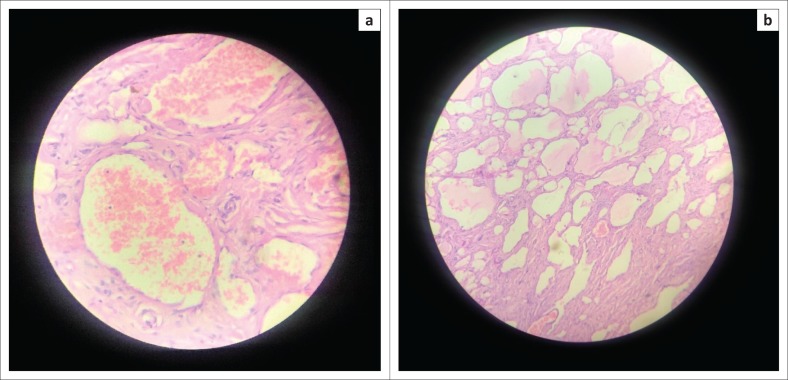
Post-operative histopathological slides of the mass with haematoxylin and eosin staining (a) and (b) show large, blood-filled cavernous vascular spaces separated by connective tissue stroma typical of cavernous haemangioma.

The patient proceeded to surgery for removal of the haemangioma prior to renal donation, and histopathological examination of the mass confirmed the diagnosis of cavernous haemangioma (Figure 6). The patient subsequently received clearance for renal donation and underwent successful renal donation as planned.

## Discussion

Hepatic haemangioma has a female predominance and may occur at any age, but more frequently occurs in the 30–50 years age group, with the mean age being 44 years.^4^ It is usually solitary and located in a subcapsular location and is more common in the right lobe of the liver, the most frequent location being the posterior segment of the right lobe. Hepatic haemangiomas are usually <3 cm in diameter.^5^ Brancatelli et al. have reported that 12% of haemangiomas demonstrate exophytic growth.^6^ However, pedunculated cases are very rare.^2,3^

The first case of a giant pedunculated haemangioma was reported by Ellis et al. in 1985. In their case, the haemangioma had presented with renal insufficiency and had been found to displace the retroperitoneal fat anteriorly simulating a retroperitoneal mass and was initially diagnosed as an adrenal mass. At surgery, the pathological examination of the mass provided the diagnosis of a giant pedunculated haemangioma.^7^

Subsequently, occasional case reports of giant pedunculated haemangiomas presenting with various symptoms or complications have been published in literature. However, these are few and far between on account of the rarity of the condition. Reports have become more commonplace in recent years, probably because of an increase in detection rates because of increasing numbers of patients undergoing MDCT scans for various indications and the development of state-of-the-art MDCT scanners.

Most hepatic haemangiomas, being small and intrahepatic, are usually asymptomatic and discovered incidentally.^1^ Giant haemangiomas may result in symptoms because of mass effect secondary to their size and may present with abdominal mass, vague abdominal pain, nausea or vomiting.^8^ Much rarer complications include congestive heart failure secondary to arteriovenous shunting in the haemangioma and thrombocytopenia with consumptive coagulopathy because of the lesion (Kasabach Meritt syndrome).^9^ Other rare complications include haemorrhage and rupture of the haemangioma.^1^

A complication specific to pedunculated haemangioma is torsion around its pedicle, subsequent to which the haemangioma undergoes vascular compromise (because of twisting of vessels in the pedicle) followed by infarction, thereby becoming symptomatic. Pain is the most frequent symptom in such cases, and it can then present as acute abdomen,^10^ mimicking a number of other conditions. There have been reports of torsion of a giant pedunculated haemangioma masquerading as acute appendicitis.^11^ Diagnosis in such cases is also challenging as the typical enhancement pattern of haemangioma may not be present because of vascular compromise.

Even otherwise, pedunculated haemangiomas of the liver are sometimes difficult to diagnose. Several conditions can present as exophytic liver masses which can provide a diagnostic challenge. Hepatic cysts, haemangiomas, focal nodular hyperplasia, hepatocellular adenomas, hepatocellular carcinoma and metastases can all present as exophytic liver masses to mention a few.^12^ Further, they may have atypical imaging appearances. Correct diagnosis of the various conditions is vital, as the management principles are drastically different for the various lesions.

Moon et al. reported a case of pedunculated hepatic haemangioma mimicking a submucosal tumour of the stomach; the lesion was symptomatic, producing epigastric discomfort with dyspepsia, and was initially suspected to be a submucosal tumour of the gastric wall on the basis of an upper gastrointestinal barium study. Computed tomography (CT) subsequently revealed a giant pedunculated haemangioma arising from the left lobe of the liver and compressing the fundus of the stomach.^13^

Giant pedunculated haemangiomas are, unlike in our case, usually symptomatic.^1^ A notable exception is the case report published by El Hajjam et al. where a giant pedunculated hepatic haemangioma was incidentally discovered in an asymptomatic patient being worked up for an anal cancer; the mass was initially suspected to be an infrahepatic peritoneal metastasis, but detailed CT and MRI examinations had revealed the characteristic enhancement pattern of a haemangioma and a thin pedicle originating from the segment IV-B of the liver. Pathological examination confirmed the diagnosis of a pedunculated hepatic haemangioma.^14^

The majority of haemangiomas are asymptomatic and do not require treatment in the absence of any definite risk factors for complications. In symptomatic cases, the treatment options include surgery, either laparoscopy or open surgery, or interventional radiology in the form of angioembolisation. The indications for surgical intervention in any haemangioma include a palpable mass, rapid growth, thrombocytopenia, symptomatic patient with abdominal discomfort or pain or complications such as rupture with intraperitoneal bleeding.^15^ However, pedunculated haemangiomas, particularly larger ones, are usually surgically resected even if asymptomatic because of the high risk of torsion and spontaneous rupture.^8^

## Conclusion

Pedunculated giant haemangioma of the liver is very rare but should always be considered as a possibility in case of an upper abdominal solid lesion in relation to the liver. It is usually symptomatic and presents with complications but may be completely asymptomatic and an incidental finding. It can be confused with a variety of other upper abdominal masses including gastric GIST as in our case. Pedunculated giant haemangiomas usually require surgical resection even if asymptomatic in view of the high risk for the development of complications. Accurate diagnosis can be difficult on the basis of imaging features but is vital in order to avoid unnecessary invasive diagnostic procedures and anxiety for the patient. The importance of the condition lies in the consequences of a misdiagnosis in terms of patient management. The onus of an accurate diagnosis lies on the radiologist.
